# Efficacy of Xiang-Sha-Liu-Jun-Zi on chemotherapy-induced nausea and vomiting: A protocol for systematic review and meta-analysis

**DOI:** 10.1097/MD.0000000000025848

**Published:** 2021-05-14

**Authors:** Hang Xiao, Liangji Liu, Shiwen Ke, Yuqin Zhang, Wenqiang Zhang, Shaobin Xiong, Wei Zhang, Jiaqing Ouyang

**Affiliations:** a Jiangxi University of Traditional Chinese Medicine; b The Affiliated Hospital of Jiangxi University of Traditional Chinese Medicine, Nanchang, Jiangxi Province, PR China.

**Keywords:** chemotherapy, nausea and vomiting, protocol, systematic review, Xiang-Sha-Liu-Jun-Zi

## Abstract

**Background:**

Cancer is the main cause of death worldwide, and chemotherapy is the basic method of treating cancer. However, chemotherapy-induced nausea and vomiting (CINV) is the most common side effect of chemotherapy, and conventional antiemetics for the treatment of CINV also have side effects. At present, a large number of randomized controlled trials have shown that Xiang-Sha-Liu-Jun-Zi (XSLJZ) can effectively treat CINV, but there is no systematic review. Therefore, this systematic review aims to discuss the effectiveness of XSLJZ in the treatment of CINV.

**Methods:**

Search for relevant documents in the Chinese and English databases, and the search time is limited to March 2021. Databases include Embase, Cochrane Library, Web of Science, PubMed, China National Knowledge Infrastructure, Chongqing VIP Information Resource Integration Service Platform, Wanfang Data, Chinese Biomedical Literature, etc. We will search the international clinical trial registration platform and the Chinese clinical trial registration platform to find ongoing and unpublished clinical trials. Randomized controlled trial of the efficacy of XSLJZ in the treatment of CINV were collected. After screening the literature according to the inclusion and exclusion criteria, two researchers independently extracted the data. The effective rate of treatment is the main outcome indicator of this study. The secondary indicators of this study include the incidence of adverse reactions and the improvement rate of quality of life. RevMan 5.3.5 software was used for statistical analysis. Grades of Recommendation, Assessment, Development, and Evaluation system will be used to evaluate the quality evidence for each outcome.

**Results:**

This study will provide the latest evidence for the treatment of CINV by XSLJZ.

**Conclusion:**

: To evaluate the efficacy of XSLJZ in the treatment of CINV.

**Unique INPLASY number:**

INPLASY202140079.

## 1 Introduction

With the progress of the world's industrialization, the aging of the population has intensified, the ecological environment has deteriorated, and the incidence and mortality of cancer have continued to increase.^[1]^ Worldwide, an estimated 19.3 million new cancer cases and almost 10.0 million cancer deaths occurred in 2020. It is estimated that by 2040, the global cancer burden will reach 28.4 million cases, an increase of 47% over 2020.^[2]^ Cancer has always been one of the leading causes of death in the world. In addition, Lung cancer, liver cancer, stomach cancer, breast cancer and colon cancer are the top five causes of cancer deaths in the world.^[3]^ However, Chemotherapy has become the basic method of treating them.^[4–8]^ Chemotherapy-induced nausea and vomiting (CINV) is a common adverse reaction during cancer treatment.^[9]^ The incidence of CINV is as high as 71.4% and 57.3%, respectively.^[10]^

CINV is not conducive to the patient's quality of life and treatment compliance, thereby hindering cancer treatment.^[11]^ According to the 2015 edition of “Anti-emetic Clinical Practice Guidelines,” the drugs used to treat CINV include 5-HT_3_ receptor antagonists, NK_1_ receptor antagonists, Dexamethasone, Phenothiazines, Benzodiazepines, H_2_ receptor antagonists, and Proton pump inhibitors. However, they have adverse effects on the nervous system, digestive system, immune system, etc.^[12]^ Traditional Chinese medicine (TCM) has multiple targets, wide curative effect, low toxic and side effects, low price, and easy access characteristics just to make up for the shortcomings of current antiemetics.^[13]^ For these reasons, TCM can be used as a new method for the treatment of CINV.

In China, TCM used to treat nausea and vomiting not only has a history of 2000 years, but also studies have shown that TCM is effective in treating CINV.^[14,15]^ Xiang-Sha-Liu-Jun-Zi (XSLJZ) created by Yunbo Ke, a doctor of Chinese medicine in the Qing Dynasty, is a classic formula of TCM.^[16]^ Modern research shows that XSLJZ can be used to treat nausea, vomiting, abdominal distension, diarrhea, and diarrhea.^[17,18]^ Therefore, it is often used to treat CINV in China. The clinical effect of XSLJZ on CINV may be achieved by up-regulating the production of ghrelin, cholecystokinin and vasoactive intestinal polypeptides and increasing the levels of these neuropeptides in the circulation.^[19]^ In addition, its possible mechanisms include eradicating HP, reducing gastric mucosal rupture, regulating gastric motility, increasing plasma motilin secretion, reducing serum gastrin levels, and enhancing smooth muscle contraction by increasing calcium levels.^[16,20]^ At present, there are a number of randomized controlled trials (RCTs) related to XSLJZ in the treatment of CINV, which all show that the efficacy is good. However, they have limitations, including small sample size, limited quality and reference values, unclear mechanisms, and no evidence-based medicine research to support it. Therefore, it is necessary for us to conduct a comprehensive meta-analysis of the clinical RCT results of XSLJZ treatment of CINV.

## 2 Methods

### 2.1 Study protocol and registration

The protocol has been registered on the INPLASY website and registration number were INPLASY202140079 (URL https://inplasy.com/inplasy-2021-4-0079/). This study will be reported in accordance with the Preferred Reporting Project Statement Guidelines for Systematic Reviews and Meta-Analysis Agreements.^[21,22]^

### 2.2 Study search

English databases include Embase, Cochrane Library, Science Network, PubMed. The Chinese database includes China National Knowledge Infrastructure, Chongqing VIP Information Resource Integration Service Platform, Wanfang Data, Chinese Biomedical Literature. Search for relevant documents in the Chinese and English databases listed above, and the search time is limited to March 2021. Search keywords include “xiangshaliujunzi,” “chemotherapy,” “nausea,” “vomiting,” “digestive reaction,” “gastrointestinal reaction.” These search terms will be accurately translated into other databases. We will also search ongoing or unpublished trials from the National Institutes of Health clinical registry Clinical Trials, International Clinical Trials Registry Platform and the Chinese clinical trial registration platform. PubMed's search strategy is shown in Table 1.

**Table 1:** Search strategy used in PubMed database.

Order	Search items
#1	(“Neoplasms”[Mesh]) OR (((((((((((Neoplasm[Title/Abstract]) undefined (Tumors[Title/Abstract])) OR (Tumor[Title/Abstract])) OR (Cancer[Title/Abstract])) OR (Cancers[Title/Abstract])) OR (Malignancy[Title/Abstract])) OR (Malignancies[Title/Abstract])) OR (Malignant Neoplasms[Title/Abstract])) OR (Malignant Neoplasm[Title/Abstract])) OR (Neoplasm, Malignant[Title/Abstract])) OR (Neoplasms, Malignant[Title/Abstract]))
#2	((“Drug Therapy”[Mesh]) OR ((((((((Therapy, Drug[Title/Abstract])) OR (Drug Therapies[Title/Abstract])) OR (Therapies, Drug[Title/Abstract])) OR (Chemotherapy[Title/Abstract])) OR (Chemotherapies[Title/Abstract])) OR (Pharmacotherapy[Title/Abstract])) OR (Pharmacotherapies[Title/Abstract]))) OR ((((((((((((Capecitabine[Title/Abstract])) OR (Fluorouracil[Title/Abstract])) OR (Tegafur Gimeracil Oteracil Potassium Capsule[Title/Abstract])) OR (Pemetrexed[Title/Abstract])) OR (Oxaliplatin[Title/Abstract])) OR (Cisplatin[Title/Abstract])) OR (Carboplatin[Title/Abstract])) OR (Nidaplatin[Title/Abstract])) OR (Vinorelbine[Title/Abstract])) OR (Etoposide[Title/Abstract])) OR (Paclitaxel[Title/Abstract]))
#3	(((“Nausea”[Mesh]) OR (((CINV[Title/Abstract]) OR (sickness[Title/Abstract])) OR (retch[Title/Abstract]))) OR (“Vomiting”[Mesh])) OR (Emesis[Title/Abstract])
#4	((((((xiangshaliujunzi[Title/Abstract]) OR (XSLJZ[Title/Abstract])) OR (xiangshaliujunzitang[Title/Abstract])) OR (XSLJZT[Title/Abstract])) OR (Chinese herbal medicine[Title/Abstract])) OR (traditional Chinese medicine[Title/Abstract])) OR (TCM[Title/Abstract])
#5	randomized controlled trial[Publication Type] OR randomized[Title/Abstract] OR placebo[Title/Abstract]
#6	#1 AND #2 AND #3 AND #4AND #5

### 2.3 Inclusion criteria for research selection

Studies that meet all of the following criteria will be included:

(1) **Type of studies.** This review will include RCTs, whether using blinding.
(2) **Type of participants.** For patients who are clearly diagnosed with cancer through pathological or cytological examination, the type of cancer, pathological type and stage are not limited. At the same time, liver and kidney indicators are basically normal, there is no contraindication to chemotherapy, and symptoms of CINV. Age and gender are not restricted.
(3) **Types of interventions.** The treatment group was treated with XSLJZ (decoction, granule, tablet and powder) combined with chemotherapy.
(4) **Type of comparators.** The control group received conventional chemotherapy.
(5) **Types of outcome measures.** The effective rate of treatment is the main outcome indicator of this study. The secondary indicators of this study include the incidence of adverse reactions and the improvement rate of quality of life.

### 2.4 Exclusion criteria for included studies

Studies that meet one of the following criteria will be excluded:

(1) Non-RCT documents.
(2) Duplicate documents.
(3) The research data is incomplete or there is no full text.
(4) Effective outcome data cannot be extracted from the literature.
(5) Documents that are not in line with the subject direction of this research.

### 2.5 Selection of studies and data extraction

**Figure 1:**
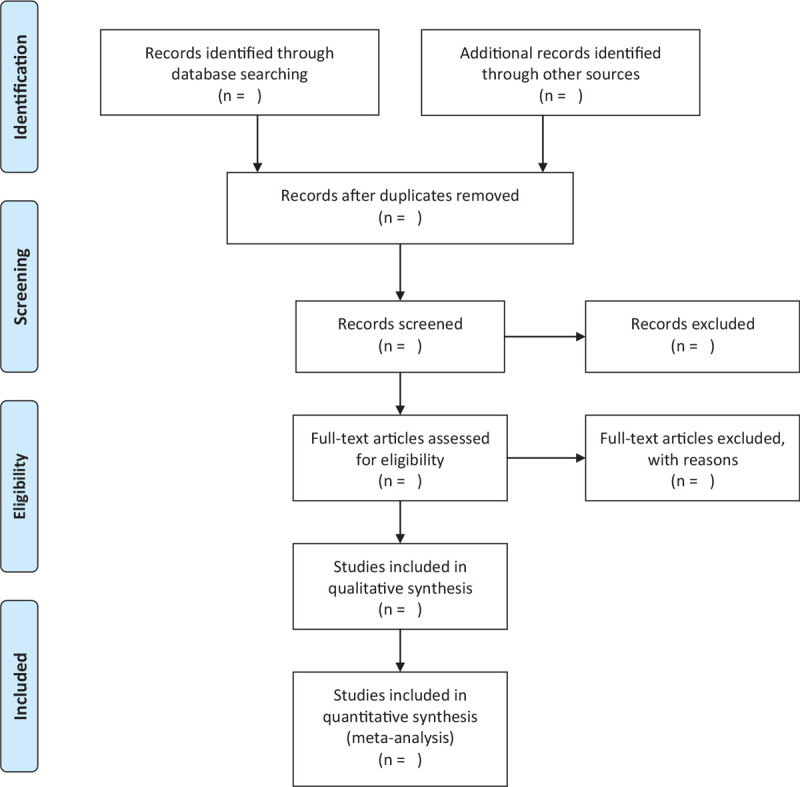
Flow chart of study selection.

First, we import all the searched literature transcripts into Endnote20.0 software, and use it to eliminate duplicate research. Secondly, after deleting the duplicate research literature, two authors independently reviewed the subject, abstract, and full text of the literature, and screened them according to the inclusion and exclusion criteria. Finally, the documents with discrepancies in the review will be reviewed and decided by the third author. The selection process will be shown through the PRISMA flowchart (Fig. 1).

The two authors independently extracted data according to a pre-designed plan and implemented cross-checking. The extracted content includes the first author, title, publication time, age, cancer type, diagnostic criteria, sample size, intervention measures, outcome indicators and related data.

### 2.6 Risk of bias assessment

The quality of all literature included in the study will be assessed using the tools of the Cochrane Intervention System Evaluation Manual.^[23]^ The categories of bias risk assessment will include: random allocation method; allocation plan concealment; blind method; completeness of result data; selective reporting of research results; other risks of bias (conflict of interest; small sample size; baseline imbalance). All the included documents will be assessed by two authors separately, and if there is any disagreement, it will be handed over to the third author for judgment. Each assessment is classified as “high risk,” “low risk” or “uncertain risk.”

### 2.7 Quantitative data synthesis and statistical methods

#### 2.7.1 Data analysis

We will use Revman 5.3 software for meta-analysis. If it is continuous data, the mean difference will be used as the combined effect statistic and its 95% confidence interval will be calculated. However, for binary data, odds ratio will be used as the combined effect statistic and its 95% confidence interval will be calculated.

#### 2.7.2 Assessment of heterogeneity

Heterogeneity assessment is to determine whether there are differences between independent studies.^[24]^ Chi-square test and *I*^*2*^ test are used to test the heterogeneity of the included literature. If *P* > .1and *I*^*2*^ < 50%, there is no heterogeneity in the independent study. The data were merged and a fixed effects model was used for meta-analysis. On the contrary, when *P* < .1 or *I*^*2*^ > 50%, independent studies are heterogeneous, and the data are merged and a random effects model is used for meta-analysis.

#### 2.7.3 Assessment of reporting biases

Generate a funnel chart and observe whether it is symmetrical to determine whether there is bias. If the graph is symmetrical, there is no bias; otherwise, there is bias.^[25]^

#### 2.7.4 Subgroup analysis

If the heterogeneity is found to be large in the heterogeneity test, a subgroup analysis is performed according to the cancer type.

#### 2.7.5 Sensitivity analysis

Revman 5.3 software was used for sensitivity analysis to evaluate the reliability of meta-analysis. If the heterogeneity is high, we need to exclude low-quality or small sample research literature, and then perform the meta-analysis again to compare the results with the non-exclusive meta-analysis. If the result is basically stable, it is considered credible.

#### 2.7.6 Assess the quality of evidence

The quality of evidence of each research article will be assessed by two authors using the Grades of Recommendation, Assessment, Development, and Evaluation system tools.^[26]^ According to the consistency, limitation, accuracy, indirectness and publication bias of the selected documents, the quality is graded, and finally divided into four grades: very low, low, medium and high.^[27]^

### 2.8 Ethics and dissemination

Since this research does not involve the personal privacy of participants, it does not require ethical approval. The research will be published in a peer-reviewed journal or related journal meeting.

## 3 Discussion

CINV is a common side effect after chemotherapy with antitumor drugs. It is usually treated with antiemetics. Obviously insufficient, antiemetics not only cause adverse reactions to the nervous system, digestive system, and immune system, but are also expensive.^[12,28,29]^ However, the traditional Chinese medicine formula XSLJZ has multiple goals, has a wide range of curative effects, small side effects, low price, and easy accessibility to make up for the shortcomings of current antiemetics.^[13]^ Studies have shown that it can act by up-regulating the production of ghrelin, cholecystokinin and vasoactive intestinal polypeptides and increasing the levels of these neuropeptides in the circulation.^[19]^ In addition, it can also eradicate HP, reduce gastric mucosal rupture, regulate gastric motility, increase plasma motilin secretion, reduce serum gastrin levels and enhance smooth muscle contraction by increasing calcium levels.^[16,20]^

At present, there is no systematic scientific evaluation of XSLJZ in the treatment of CINV, but RCTs have shown its effectiveness, so this article aims to provide evidence-based medical evidence for its efficacy. Of course, this study still has limitations: first, the poor quality of the retrieved literature may affect the evaluation results; second, the literature has Chinese and English language restrictions, which may lead to incomplete literature retrieval. Third, there may be documents whose authors cannot be contacted, resulting in incomplete data results. Therefore, more high-quality RCTs and research mechanisms are needed to confirm their effectiveness in order to more objectively evaluate the effectiveness of XSLJZ in the treatment of CINV.

## Author contributions

**Conceptualization:** Hang Xiao and Liangji Liu.

**Data curation:** Hang Xiao, Yuqin Zhang, and Wenqiang Zhang.

**Formal analysis:** Shiwen Ke, Shaobin Xiong, Jiaqing Ouyang.

**Methodology:** Liangji Liu, Shiwen Ke, and Wenqiang Zhang.

**Software:** Yuqin Zhang, Shaobin Xiong, and Hang Xiao.

**Supervision:** Hang Xiao and Liangji Liu.

**Writing – original draft:** Hang Xiao, Wei Zhang, and Yuqin Zhang.

**Writing – review & editing:** Hang Xiao, Wenqiang Zhang, Shaobin Xiong, and Wei Zhang.
